# SGLT2 Inhibitor Empagliflozin and DPP4 Inhibitor Linagliptin Reactivate Glomerular Autophagy in *db/db* Mice, a Model of Type 2 Diabetes

**DOI:** 10.3390/ijms21082987

**Published:** 2020-04-23

**Authors:** Anton I. Korbut, Iuliia S. Taskaeva, Nataliya P. Bgatova, Natalia A. Muraleva, Nikolai B. Orlov, Maksim V. Dashkin, Anna S. Khotskina, Evgenii L. Zavyalov, Vladimir I. Konenkov, Thomas Klein, Vadim V. Klimontov

**Affiliations:** 1 Research Institute of Clinical and Experimental Lymphology—Branch of the Institute of Cytology and Genetics, Siberian Branch of Russian Academy of Sciences (RICEL—Branch of IC&G SB RAS), Timakov Str. 2, 630060 Novosibirsk, Russia; 2 Institute of Cytology and Genetics, Siberian Branch of Russian Academy of Sciences (IC&G SB RAS), Lavrentjev Prospect 10, 630090 Novosibirsk, Russia; 3 Department of Cardiometabolic Diseases Research, Boehringer Ingelheim Pharma GmbH & Co. KG, Birkendorfer Str. 65, 88397 Biberach, Germany

**Keywords:** autophagy, podocyte, type 2 diabetes, diabetic nephropathy, empagliflozin, linagliptin

## Abstract

Recent data have indicated the emerging role of glomerular autophagy in diabetic kidney disease. We aimed to assess the effect of the SGLT2 inhibitor empagliflozin, the DPP4 inhibitor linagliptin, and their combination, on glomerular autophagy in a model of type 2 diabetes. Eight-week-old male *db/db* mice were randomly assigned to treatment with empagliflozin, linagliptin, empagliflozin–linagliptin or vehicle for 8 weeks. Age-matched non-diabetic *db/+* mice acted as controls. To estimate glomerular autophagy, immunohistochemistry for beclin-1 and LAMP-1 was performed. Podocyte autophagy was assessed by counting the volume density (Vv) of autophagosomes, lysosomes and autolysosomes by transmission electron microscopy. LC3B and LAMP-1, autophagy markers, and caspase-3 and Bcl-2, apoptotic markers, were evaluated in renal cortex by western blot. Vehicle-treated *db/db* mice had weak glomerular staining for beclin-1 and LAMP-1 and reduced Vv of autophagosomes, autolysosomes and lysosomes in podocytes. Empagliflozin and linagliptin, both as monotherapy and in combination, enhanced the areas of glomerular staining for beclin-1 and LAMP-1 and increased Vv of autophagosomes and autolysosomes in podocytes. Renal LC3B and Bcl-2 were restored in actively treated animals. LAMP-1 expression was enhanced in the empagliflozin group; caspase-3 expression decreased in the empagliflozin–linagliptin group only. Mesangial expansion, podocyte foot process effacement and urinary albumin excretion were mitigated by both agents. The data provide further explanation for the mechanism of the renoprotective effect of SGLT2 inhibitors and DPP4 inhibitors in diabetes.

## 1. Introduction

Diabetic nephropathy is the most common cause for end-stage renal disease worldwide [1]. The pathogenesis of diabetic nephropathy is being studied extensively. A number of studies have identified podocyte injury as a cornerstone in pathogenesis of diabetic kidney disease [2,3]. Podocytes are terminally differentiated cells that wrap the glomerular capillary loops from outside and form slit diaphragms by podocyte foot processes (FPs). Diabetes-induced podocyte injury results in several phenotypic changes, including effacement of FPs, hypertrophy, detachment, death, and/or dedifferentiation. In its turn, the loss or damage of podocytes causes dysfunction of the filtration barrier and increases albuminuria [2].

Some recent studies have indicated an emerging role of autophagy downregulation in diabetic podocytopathy [4,5,6]. Autophagy is a cellular recycling process involving self-degradation and reconstruction of damaged organelles and proteins [6]. The process is vital for highly differentiated post-mitotic cells, such as neurons and podocytes [7]. A growing body of evidence indicates a critical role of autophagy in maintaining podocyte integrity and renal function [6]. Mice with podocyte-specific deletion of autophagic regulators, such as class III PI3K vacuolar protein sorting 34 (Vps34) and the Atg5 gene, develop early proteinuria, progressive glomerulosclerosis, and renal failure [6]. Accordingly, autophagy is considered a potential therapeutic target for renal protection [8,9].

Sodium-glucose cotransporter-2 (SGLT2) inhibitors and dipeptidylpeptidase-4 (DPP4) inhibitors are promising antidiabetic agents introduced into clinical practice in the last decade. The antihyperglycemic effect of SGLT2 inhibitors is mediated by increment of glucosuria, while DPP4 inhibitors realize their activity through an increase in the half-life of incretin hormones. Both SGLT2 and DPP4 inhibitors demonstrated renal protective activity in large-scaled randomized clinical trials. Specifically, in the Empagliflozin Cardiovascular Outcome Event Trial in Type 2 Diabetes Mellitus Patients (EMPA-REG OUTCOME trial) SGLT2 inhibitor empagliflozin reduced the risk of progression to macroalbuminuria, a doubling of the serum creatinine, initiation of renal replacement therapy or death from renal disease in patients with type 2 diabetes [10]. In the Cardiovascular and Renal Microvascular Outcome Study with Linagliptin in Patients with Type 2 Diabetes Mellitus (CARMELINA study), the DPP4 inhibitor linagliptin retarded the albuminuria progression in patients with type 2 diabetes and high cardiovascular risk [11]. It is assumed that double inhibition of SGLT2 and DPP4 may have advantages in terms of renoprotection [12,13].

The mechanisms of protective action of SGLT2 inhibitors and DPP4 inhibitors on diabetic kidney are still a matter for debate. SGLT2 inhibitors have been shown to enhance sodium excretion and suppress glomerulosclerosis and interstitial fibrosis, inflammation and oxidative stress in diabetic kidney [14,15,16]. In addition, DPP4 inhibitors have been showed to reduce glomerular hypertension and hyperfiltration [17,18,19]. Moreover, empagliflozin has been shown to activate autophagy in tubular cells [20,21]. However, the effect of SGLT2 and DPP4 inhibition on glomerular autophagy has not been studied yet [22].

Therefore, we aimed to assess the effect of SGLT2 inhibitor empagliflozin, DPP4 inhibitor linagliptin, and a combination of both agents on glomerular autophagy in a model of type 2 diabetic kidney disease.

## 2. Results

### 2.1. Body Composition and Biochemical Parameters

Diabetic *db/db* mice had become obese and hyperglycemic prior to the start of experiment. When compared to *db/+* controls, 8-week-old *db/db* mice demonstrated increase in their fat mass and reduction of lean mass and water content (Table 1). In vehicle-treated mice, these changes remained stable throughout the experiment. In actively treated animals, especially in empagliflozin groups, a further increase in the body weight and fat mass was observed.

Diabetic mice demonstrated increased plasma glucose, fructosamine, glycated albumin, leptin, insulin and PAI-1 levels; meanwhile, ghrelin concentrations were decreased significantly (Table 2). Groups of animals were heterogeneous for plasma glucagon levels at week 8 and week 16. Severe hyperglycemia persisted throughout the experiment in vehicle-treated and linagliptin-treated *db/db* mice.

Under the treatment with empagliflozin or empagliflozin–linagliptin combination, *db/db* mice demonstrated improvement in their glycemic status, which was documented by the reduction in their plasma glucose, fructosamine and glycated albumin (empagliflozin: *p* = 0.02, *p* = 0.02, and *p* = 0.02, respectively; combination: *p* = 0.03, *p* = 0.046, and *p* = 0.046, respectively, vs. week 8). Meanwhile, no significant changes in glycemic control were observed in the linagliptin group. The levels of insulin, glucagon, ghrelin and PAI-1 did not change significantly throughout the experiment in all groups (*p* > 0.05 week 16 vs. week 8). However, plasma leptin levels were increased significantly in the empagliflozin group and tended to increase in the empagliflozin–linagliptin group (*p* = 0.03 and *p* = 0.08, respectively, week 16 vs. week 8). Meanwhile, empagliflozin–linagliptin-treated *db*/*db* mice demonstrated lower plasma levels of glucagon as compared with vehicle-treated *db/db* mice (*p* = 0.04).

### 2.2. Albuminuria and Renal Function 

A markedly increased albuminuria was detected in all diabetic mice groups at the start of experiment (all *p* < 0.001, Table 3). Administration of empagliflozin, linagliptin, or both agents decreased albumin excretion (*p* = 0.007, *p* = 0.03, and *p* = 0.04, respectively, vs. week 8). No significant changes in the levels of plasma creatinine were detected throughout the study.

### 2.3. Renal Hypertrophy, Glomerular and Podocyte Morphology

**Figure 1:**
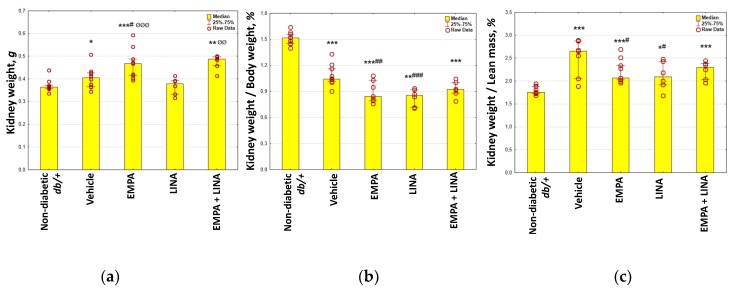
Parameters of kidney weight in non-diabetic *db/+* mice and diabetic *db/db* mice: (**a**) kidney weight, g; (**b**) kidney weight/body weight ratio, %; (**c**) kidney weight/lean mass ratio, %. EMPA, empagliflozin-treated *db/db* mice; LINA, linagliptin-treated *db/db* mice; EMPA+LINA, empagliflozin–linagliptin-treated *db/db* mice. Data are presented as a bar graph (median, lower and upper quartile) with individual data set (dots); * *p* < 0.05, ** *p* < 0.01, *** *p* < 0.001 vs. non-diabetic control (*db/+* mice), ^#^ *p* < 0.05, ^##^ *p* < 0.01, ^###^ *p* < 0.001 vs. vehicle-treated *db/db* mice, ^ØØ^ *p* < 0.01, ^ØØØ^ *p* < 0.001 vs. linagliptin-treated *db/db* mice.

Vehicle-treated *db/db* mice demonstrated increase in their kidney weight and kidney weight/lean mass ratio, whereas kidney weight/body weight ratio was decreased (*p* = 0.048, *p* = 0.0007 and *p* = 0.00002 vs. non-diabetic *db/+* mice, respectively, Figure 1). The kidney weight/body weight and kidney weight/lean mass ratio was lower in empagliflozin and linagliptin-treated mice as compared to vehicle-treated animals (all *p* < 0.05).

**Figure 2:**
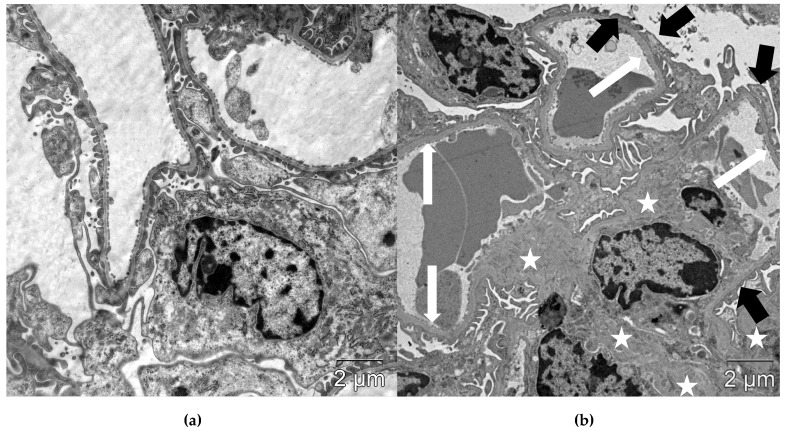
Glomerular structure in non-diabetic *db/+* mice (**a**) and vehicle-treated diabetic *db/db* mice (**b**). Diabetic mice demonstrated thickening of GBM (white arrows), effacement of podocyte FPs (black arrows) and mesangial expansion (white stars). Scale bar 2 µm. TEM.

Vehicle-treated *db/db* mice demonstrated typical morphological signs of diabetic nephropathy, including mesangial expansion, thickening of glomerular basement membrane (GBM), and effacement of podocyte foot processes (FPs). These changes were quantified by morphometric analysis (Table 4, Figure 2).

The structural changes in the kidneys were mitigated by empagliflozin, linagliptin, and combined treatment. Specifically, fractional mesangial volume and mean GBM width were reduced (*p* = 0.0008 and *p* = 0.02 for empagliflozin; *p* = 0.002 and *p* = 0.04 for linagliptin; *p* = 0.03 and *p* = 0.04 for combination, respectively). The mean width of podocyte FPs decreased in empagliflozin-treated, linagliptin-treated and empagliflozin–linagliptin-treated mice (*p* = 0.005, *p* = 0.02, and *p* = 0.004, respectively); accordingly, N_A_ of FPs increased in these groups (*p* = 0.03, *p* = 0.04, and *p* = 0.03, respectively).

### 2.4. Expression of Autophagy and Apoptosis Regulators in the Renal Cortex

**Figure 3:**
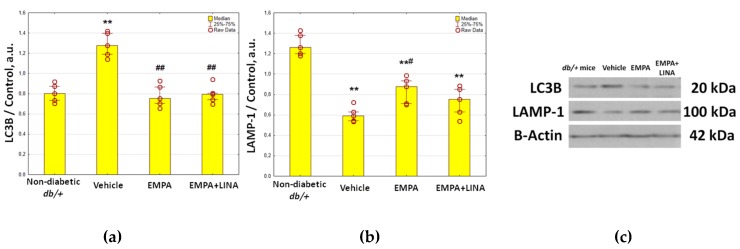
Protein expression of LC3B, an autophagosome marker (**a**), and LAMP-1, a lysosome marker (**b**), in the renal cortex from non-diabetic *db/+* mice and diabetic *db/db* mice treated with vehicle, empagliflozin, or empagliflozin–linagliptin; (c) representative immunoblots of LC3B, LAMP-1 and β-actin as the control. Data are presented as a bar graph (median, lower and upper quartile) with individual data set (dots); a.u., arbitrary unit; ** *p* < 0.01 vs. non-diabetic *db/+* mice; ^#^ *p* < 0.05, ^##^ *p* < 0.01 vs. vehicle-treated *db/db* mice (Mann–Whitney U-test).

Western blotting analysis showed protein expression of LC3B, an autophagosome marker, to be significantly elevated in renal cortex in vehicle-treated *db/db* mice compared to controls (*p*=0.008, Figure 3). We found decrease in the protein expression of LC3B in *db/db* mice treated with empagliflozin and the combination of empagliflozin and linagliptin (both *p* = 0.008 vs. vehicle group). Protein expression of LAMP-1, a lysosomal marker, was reduced significantly in vehicle-treated *db/db* mice when compared to non-diabetic *db/+* mice (*p* = 0.008, Figure 3). In the empagliflozin group, the amount of LAMP-1 was restored (*p* = 0.03 vs. vehicle-treated *db/db* mice). Although empagliflozin–linagliptin-treated *db/db* mice demonstrated similar changes in LAMP-1 protein expression, statistical significance was not achieved (*p* = 0.15 vs. vehicle-treated *db/db* mice).

**Figure 4:**
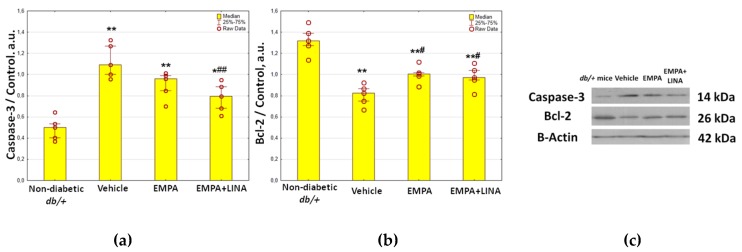
Protein expression of caspase-3, a regulator of apoptosis **(a),** and Bcl-2, an anti-apoptotic protein **(b)** in the renal cortex from non-diabetic *db/+* mice and diabetic *db/db* mice treated with vehicle, empagliflozin, and empagliflozin–linagliptin; **(c)** representative immunoblots of caspase-3, Bcl-2 and β-actin as the control. Data are presented as a bar graph (median, lower and upper quartile) with individual data set (dots); a.u., arbitrary unit; * *p* < 0.05, ** *p* < 0.01 vs. non-diabetic *db/+* mice; *^#^ p* < 0.05,*^##^ p* < 0.01 vs. vehicle-treated *db/db* mice (Mann–Whitney *U*-test).

The levels of caspase-3 expression, an apoptosis marker, were elevated markedly in vehicle-treated *db/db* mice (*p* = 0.008, Figure 4). The expression of caspase-3 tended to be lower in empagliflozin-treated *db/db* mice (*p* = 0.095 vs. vehicle-treated *db/db* mice); it was significantly lower in *db/db* mice treated with empagliflozin and linagliptin (*p* = 0.008 vs. vehicle-treated *db/db* mice).

The expression of Bcl-2, an anti-apoptotic protein, was lower in the kidneys of vehicle-treated *db/db* mice as compared to *db/+* controls (*p* = 0.008, Figure 4). The *db/db* mice treated with empagliflozin and empagliflozin–linagliptin demonstrated increase in Bcl-2 expression when compared to vehicle-treated animals (*p* = 0.02 and *p* = 0.046, respectively).

### 2.5. Glomerular Expression of the Autophagy Markers

**Figure 5:**
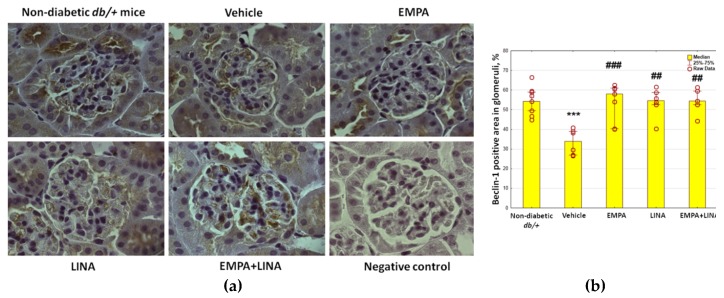
Glomerular beclin-1 expression in non-diabetic *db/+* mice and diabetic *db/db* mice. (**a**) IHC-pictures of glomeruli of non-diabetic *db/+* mice and *db/db* mice treated by vehicle, empagliflozin, linagliptin, or empagliflozin–linagliptin; magnification ×400; **(b)** Quantitative analysis of beclin-1-positive area in glomeruli. Data are presented as a bar graph (median, lower and upper quartile) with individual data set (dots); *** *p* < 0.001 vs. non-diabetic *db/+* mice; *^##^ p* < 0.01,*^###^ p* < 0.001 vs. vehicle-treated *db/db* mice (Mann–Whitney *U*-test).

The glomerular expression of beclin-1 and LAMP-1, two markers attributed to autophagy, was assessed by immunohistochemistry (IHC). Vehicle-treated *db/db* mice demonstrated a reduction of beclin-1-positive areas in glomeruli (*p* = 0.00002 vs. non-diabetic *db/+* mice). This reduction was mitigated by empagliflozin, linagliptin, or empagliflozin–linagliptin treatment (*p* = 0.0003, *p* = 0.001 and *p* = 0.001, respectively vs. vehicle-treated *db/db* mice, Figure 5).

**Figure 6:**
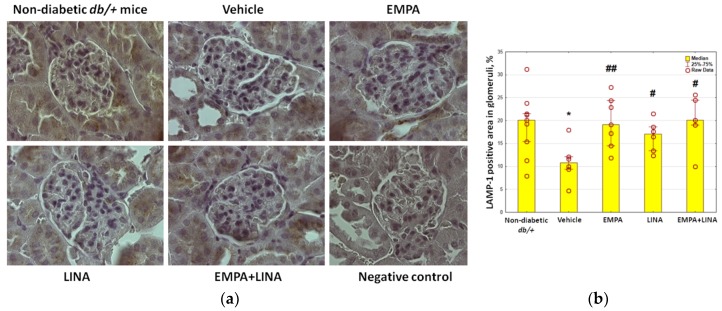
Glomerular LAMP-1 expression in non-diabetic *db/+* mice and diabetic *db/db* mice. (**a**) IHC-pictures of glomeruli of non-diabetic *db/+* mice and *db/db* mice treated by vehicle, empagliflozin, linagliptin, or empagliflozin–linagliptin; magnification ×400; **(b)** Quantitative analysis of LAMP-1-positive areas in glomeruli. Data are presented as a bar graph (median, lower and upper quartile) with individual data set (dots); * *p* < 0.05 vs. non-diabetic *db/+* mice; *^#^ p* < 0.05,*^##^ p* < 0.01 vs. vehicle-treated *db/db* mice (Mann–Whitney *U*-test).

The area of the staining for LAMP-1 was reduced in vehicle-treated *db/db* mice as compared to non-diabetic *db/+* controls (*p* = 0.01). Administration of empagliflozin, linagliptin or both agents preserved this reduction (*p* = 0.002, *p* = 0.04 and *p* = 0.01 vs. vehicle-treated *db/db* mice respectively, Figure 6).

### 2.6. Autophagy in Podocytes

The volume density (Vv) of autophagosomes, autolysosomes and lysosomes in podocytes was assessed from TEM images. Vehicle-treated diabetic *db/db* mice had reduced Vv of autophagosomes in podocytes (*p* = 0.00002); administration of empagliflozin, linagliptin, or both agents increased Vv of autophagosomes significantly (*p* = 0.00004, *p* = 0.0003, and *p* = 0.0005, respectively, Table 5).

There was a reduction in the count of autolysosomes in vehicle-treated *db/db* mice (*p* = 0.00002, Table 5). Nevertheless, diabetic *db/db* mice treated with empagliflozin, linagliptin or the combination of both demonstrated an increase in Vv of autolysosomes (*p* = 0.0003, *p* = 0.0005, and *p* = 0.0005, respectively). The changes in the count of lysosomes under these treatments were similar (*p* = 0.002, *p* = 0.04, and *p* = 0.0005, respectively).

### 2.7. Relationships between Autophagy Intensity, Glomerular Morphology and Biochemical Parameters

In *db/+* and *db/db* mice, both beclin-1 and LAMP-1 correlated positively with Vv of autophagosomes and autolysosomes in podocytes (Table 6). Additionally, the area of the staining for LAMP-1 and a protein amount of LAMP-1 demonstrated a correlation with Vv of the lysosomes. The Vv of autophagosomes and autolysosomes correlated positively with renal LAMP-1 and Bcl-2. However, Vv of autophagosomes, autolysosomes and lysosomes correlated negatively with renal LC3B and caspase-3.

Kidney weight/lean mass ratio, considered as an indicator of renal hypertrophy, was found to correlate negatively with Vv of autolysosomes and lysosomes in podocytes (Table 7). Mesangial fractional volume correlated negatively with all studied autophagy parameters. A similar pattern of relationships was demonstrated for GBM width. The correlations between metrics of podocyte FPs and autophagy indicators were also revealed.

Renal cortex expression of caspase-3, LAMP-2 and Bcl-2 was associated with kidney weight/lean mass ratio (Table 8). The mesangial fractional volume and GBM width correlated positively with renal LC3B and caspase-3. Contrary, renal LAMP-1 and Bcl-2 demonstrated negative correlations with mesangial fractional volume and GBM width. Renal LC3B and caspase-3 were associated with the changes in podocyte FPs.

There were negative correlations between indicators of autophagy and glycemic control parameters. Specifically, plasma fructosamine and glycated albumin correlated with renal LAMP-1 (*r* = −0.65, *p* = 0.002 and *r* = −0.42, *p* = 0.007 respectively), glomerular beclin−1 (*r* = −0.34, *p* = 0.04 for both) and LAMP−1 (*r* = −0.41, *p* = 0.008; *r* = −0.42, *p* = 0.007), podocyte Vv of autophagosomes (*r* = −0.34, *p* = 0.03; *r* = −0.32, *p* = 0.046), autolysosomes (*r* = −0.34, *p* = 0.03; *r* = −0.31, *p* > 0.05) and lysosomes (*r* = −0.36, *p* = 0.02; *r* = −0.33, *p* = 0.04). No correlations were found with plasma leptin, insulin, ghrelin and PAI-1. However, plasma glucagon concentrations showed negative correlations with glomerular LAMP-1 (*r* = −0.39, *p* = 0.03), Vv of autophagosomes (*r* = −0.44, *p* = 0.005) and lysosomes (*r* = −0.38, *p* = 0.02). 

Renal caspase-3 demonstrated strong positive correlations with fructosamine and glycated albumin levels (*r* = 0.91, *p* < 0.0001 and *r* = 0.89, *p* < 0.0001, respectively); renal Bcl-2 correlated with these parameters negatively (*r* = −0.66, *p* = 0.001; *r* = −0.67, *p* = 0.001).

We found no correlations between autophagy and apoptosis parameters and plasma creatinine levels. Meanwhile, urinary albumin-to-creatinine ratio (UACR) correlated negatively with renal LAMP-1 (*r* = −0.76, *p* = 0.001), Bcl-2 (*r* = −0.81, *p* < 0.0001), glomerular beclin-1 (*r* = −0.44, *p* = 0.006) and Vv of autophagosomes (*r* = −0.47, *p* = 0.002), autolysosomes (*r* = −0.48, *p* = 0.002) and lysosomes (*r* = −0.53, *p* = 0.0004) in podocytes. There were positive correlations between UACR and renal LC3B (*r* = 0.50, *p* = 0.03) and caspase-3 expression (*r* = 0.66, *p* = 0.002).

## 3. Discussion

In this study, we have demonstrated, for the first time, that the SGLT2 inhibitor empagliflozin, the DPP4 inhibitor linagliptin, and a combination of these agents, could reactivate glomerular autophagy in *db/db* mice, a model of type 2 diabetes. The effect is associated with mitigation of renal hypertrophy, an improvement in glomerular morphology, including a decrease in the severity of podocytopathy, and a slowdown in the growth of albuminuria. 

### 3.1. Suppression of Renal Autophagy in Diabetes: Markers and Mechanisms

The suppression of macroautophagy in *db/db* diabetic mice was documented by the reduction of glomerular IHC-staining for beclin-1 and LAMP-1, two autophagy markers, and decrease in the volume density of the autophagic compartments (autophagosomes, autolysosomes, and lysosomes) in the podocytes. We have also revealed the reduced expression of LAMP-1 in the renal cortex by western blotting. The results are in agreement with the data from other researches recorded downregulation of autophagy in diabetic kidneys [4,23,24,25,26].

The data on the enhanced expression of LC3B in the renal cortex, obtained by western blot, at first glance are out of the picture. The LC3 molecule is commonly considered a marker of autophagosomes, although it could also be detected in phagophores [27]. LC3-II is generated by the conjugation of cytosolic LC3-I to phosphatidylethanolamine on the surface of nascent autophagosomes. Accordingly, LC3-II is relatively specifically associated with autophagosomes and autolysosomes [28]. In previous studies, both a decrease in LC3-II/LC3-I ratio [29,30] and an increase in LC3-I/II expression [31] in the kidneys of *db/db* mice was reported. In podocyte cell culture, the expression of LC3-II was increased under high glucose condition [32]. Similarly, an increased LC3B/LC3A ratio was found in high-fat-diet-induced, obesity-related glomerulopathy in mice [33]. Increased LC3-II expression was previously described in tubular epithelial cells in diabetes [34]. Accumulation of LC3-II could be observed when the fusion of autophagosomes with lysosomes was interrupted by chemical agents [27]. It was demonstrated that endogenous LC3-positive puncta become larger in cells where autophagosome formation is abrogated and are prominent even when LC3-II is not formed. This occurs even with transient and incomplete inhibition of autophagosome biogenesis. This phenomenon is due to LC3-I sequestration to p62 aggregates, which accumulate when autophagy is impaired [28]. In this study, we found negative correlations between LC3B levels in the renal cortex and the volume density of lysosomes and autolysosomes in the podocytes. Taking into account a decrease in V_V_ of autophagosomes and lysosomes, as well as a decrease in the renal expression of other markers of autophagy, we can assume that an increase in LC3B protein could be attributed to compromised autophagy in diabetic kidney.

The mechanisms of autophagy suppression in diabetic kidneys remain a matter of debate. Hyperglycemia was considered an autophagy suppressor in the studies with cultured podocytes [24,35], glomerular endothelial cells [36] and mesangial cells [37]. Kidney macroautophagy is regulated by mammalian target of rapamycin (mTOR) [8,38,39], AMP-activated protein kinase (AMPK) [8], SIRT1 [8], Wnt/β-catenin [40], and TGF-β [41] signaling pathways. The downregulation of glomerular autophagy under hyperglycemic condition is considered a result of activation of mTOR pathway [8,42] or suppression of AMPK activity and SIRT1 signaling [37,43,44]. In our study, glomerular autophagy was related negatively to parameters of glycemic status, fructosamine and glycated albumin. As it has been demonstrated previously, advanced glycation end-products impair autophagic flux in the podocytes via mTOR activation and inhibition of the nuclear translocation and activity of the pro-autophagic transcription factor EB (TFEB) [45]. In cultured mesangial cells, advanced glycation end-products inhibited autophagy via the RAGE/STAT5 axis [46]. Therefore, hyperglycemia and related activation of non-enzymatic glycation could be a cornerstone in glomerular autophagy suppression.

In this study, we used leptin receptor-defective *db/db* mice as a model of type 2 diabetes. In these mice, we found extremely high levels of leptin, which can be explained by obesity and defective leptin signaling. The recent data indicate that leptin plays an important role in the neuroendocrine control of autophagy. It was demonstrated that intravenous or intraperitoneal administration of recombinant leptin induced autophagy in mouse peripheral tissues, including skeletal muscle, heart and liver. Moreover, leptin stimulated canonical autophagy in cultured human or mouse cell lines, a phenomenon that was coupled to the activation of AMPK, as well as the inhibition of mTOR, and this was confirmed by autophagic flux measurements [47]. Thereby, it can be assumed that the defect of leptin receptor is another factor contributing to the suppression of autophagy in *db/db* mice.

The recorded high insulin levels in plasma and severe hyperglycemia in *db/db* mice undoubtedly indicate insulin resistance, which is consistent with the literature [48]. Intrinsic interactions between insulin signaling and podocyte autophagy have been proposed. The recent findings suggested that downregulation of podocyte autophagy, observed after long-term exposure to high glucose, results from suppressed sensitivity to insulin [49]. Podocytes express all the elements of the insulin-signaling cascade, such as functional insulin receptor substrate-1 and insulin receptor, and are capable of increasing their glucose uptake when they are stimulated with insulin through glucose transporters, primarily glucose transporter type 4 (GLUT4) [50]. Glomeruli from mice with podocyte-specific knockout of GLUT4 are protected from diabetes-induced hypertrophy, mesangial expansion and albuminuria, and fail to activate the mTOR pathway [51]. The latter is activated in podocytes in diabetic conditions that decrease autophagy activity [42]. Interestingly, rapamycin-induced autophagy increased the glucose uptake in the podocytes and phosphorylated insulin receptor, which caused an increase in insulin sensitivity [24].

In addition to insulin, the activity of autophagy in diabetes can be modified by other hormonal regulators. We recorded a significant decrease in concentrations of ghrelin in the plasma of *db/db* mice. Ghrelin is a proven enhancer of autophagy, mediating its effect through the activation of AMPK [52]. Additionally, PAI-1 is considered an autophagy activator in immune cells [53]. In a model of vinyl-chloride-induced renal fibrosis, activation of autophagy corresponded to increased PAI-1 expression, while expression of PAI-1 gene in HK-2 cell culture was increased under beclin-1 siRNA exposure [54]. Nevertheless, in our study we failed to find any relationship between plasma PAI-1 levels and autophagy parameters.

Glucagon is shown to regulate autophagy by increasing the number and fragility of lysosomes and apoptotic vacuoles. These effects were primary described in liver and suggested to be organ-specific [55]. In our study, plasma glucagon levels correlated negatively with glomerular LAMP-1 and volume density of autophagosomes and lysosomes in the podocytes. These results do not prove causality; the role of glucagon in regulation of renal autophagy in diabetes requires further research.

### 3.2. Renal Autophagy Suppression in Pathophysiology of Diabetic Kidney Disease

In our study, the suppression of autophagy was related to severity of the classical signs of diabetic nephropathy, including mesangial expansion, GBM thickening and podocytopathy. It was suggested that the presence or activation of autophagy could play a protective role in human podocytes against high glucose-induced insulin resistance and cell injury [24]. In its turn, downregulation of autophagy is critical for podocyte survival in hyperglycemic conditions [49]. In the study with autophagy-deficient models of diabetes, blocking of autophagy in podocytes or glomerular endothelial cells caused podocytopathy and leaky glomerular filtration barriers [36]. The deficiency of autophagy activation in KkAy diabetic mice resulted in more severe proteinuria and impaired renal function [26]. In agreement with these data, we found an inverse association between morphometric parameters of glomerular autophagy, podocytopathy and albuminuria in the model of type 2 diabetes.

Apoptosis is considered another contributor to podocyte depletion in diabetes [42]. In this study, we investigated the expression of caspase-3, an apoptosis regulator, and Bcl-2, an anti-apoptotic protein, in the renal cortex of *db/db* mice. We found increased protein expression of caspase-3; meanwhile, Bcl-2 expression was diminished. Thus, we recorded reciprocal changes in the levels of autophagy and apoptosis markers. It was demonstrated recently that autophagy can regulate apoptosis following molecular interactions between the key proteins of these processes, including members of Bcl-2 family, autophagy proteins, and caspases [56]. Firstly, beclin-1 can bind to Bcl-2, which results in inhibition of autophagy [56]. Contrary, release of beclin-1 from the complex with Bcl-2 and phosphorylation of beclin-1 or Bcl-2 promote autophagy [27]. In addition, cleavage of beclin-1 by caspase-3 inactivates autophagy and promotes apoptosis [57]. This could be an explanation for the concomitant increase in caspase-3 and decrease in beclin-1 expression recorded in our study.

### 3.3. Reactivation of Autophagy: a Missing Link in the Mechanism of Renoprotection under SGLT2 and DPP4 Inhibition?

In this work, we demonstrate that empagliflozin and linagliptin, both as monotherapy and in combination, reactivate glomerular autophagy in *db/db* diabetic mice. In the previous studies, empagliflozin restored autophagy in the tubular cells in a model of streptozotocin-induced diabetes [20], and in the heart in a model of myocardial infarction and type 2 diabetes [58]. Canagliflozin, another SGLT2 inhibitor, activated autophagy in immune cells [29]. A negative correlation between beclin-1 and DPP4 activity was observed in patients with coronary heart disease [59]. The DPP4 inhibitors vildagliptin and sitagliptin improved cardiac function after myocardial infarction through activation of autophagy in the rodent models of diabetes [60,61]. Sitagliptin activated autophagy in cardiac muscle in Zucker diabetic fatty rats, in high-glucose cultured embryonic heart myoblastic (H9c2) cells [59] and in the liver of *ob/ob* mice, a mouse model of genetic obesity and diabetes [62].

According to our data, empagliflozin, both alone and in combination with linagliptin, contributed to preventing changes in the renal expression of apoptosis regulators, caspase-3 and Bcl-2. In the previous studies, empagliflozin have demonstrated decreased expression of pro-apoptotic proteins in cell cultures of human renal proximal tubular cells (hRPTCs) [20] and in the kidney in streptozotocin-induced diabetic rats [63]. Taking into account the close relationships in the regulation of autophagy and apoptosis, normalization of the expression of apoptosis regulators could also contribute to the reactivation of autophagy under empagliflozin treatment.

It is well known that both SGLT2 and DPP4 inhibitors generate glucose-dependent and glucose-independent effects in the targeted organs [14,15,16,17,18,19]. In our study, autophagy was reactivated by both empagliflozin and linagliptin, while glucose levels were reduced, but not to the normal range, only under empagliflozin administration. We did not observe antihyperglycemic effects of lingagliptin in *db/db* mice in our study, although we proved renal benefits of the agent. Similarly, in previous studies, linagliptin ameliorated glomerular changes in *db/db* mice, despite of the lack of antihyperglycemic activity [64,65]. This suggests a glucose-independent mechanism of autophagy reactivation. It was demonstrated that vildagliptin suppressed Beclin-1–Bcl-2 interaction and increased both LC3-II protein level and autophagosomes in the diabetic heart [60]. In the liver of *ob/ob* mice, sitagliptin increased the levels of phosphorylation of AMPK and decreased those of mTOR [62]. The activation of the AMPK signaling pathway in immune cells was proven for canagliflozin [29]. Further identification of the molecular mechanisms for autophagy activation under SGLT2 and DPP4 inhibition is a challenge for future research.

## 4. Materials and Methods

### 4.1. The Design of the Study

The study was carried out with *db/db*-specific pathogen-free mice (BKS.Cg- Dock7^m^+/+Lepr^db^/J, stock #000642) obtained from Jackson Laboratory (Bar Harbor, Maine, CA, USA). The animals were housed with a regular 12/12-hour light/dark cycle (lights on 02:00 a.m.), a constant room temperature of 23 ± 2°C, and a relative humidity of 45 ± 10%. The 3-week-old mice were separated from mothers and housed in OptiMICE individually ventilated cages (Animal Care Systems, Centennial, CO, USA), in groups of two to three animals per cage with ad libitum food (Ssniff, Germany) and water.

**Figure 7:**
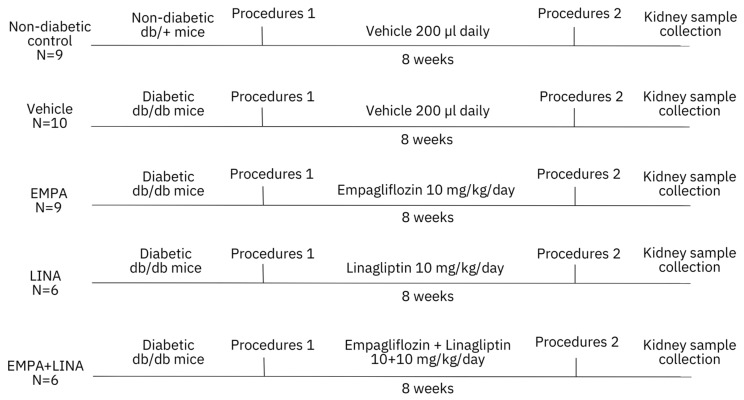
Flowchart of the study. The experiment was started with 8-week-old non-diabetic *db+* and diabetic *db/db* mice. Diabetic mice were randomly assigned for treatment with a vehicle, empagliflozin, linagliptin, or combination of empagliflozin and linagliptin. The total duration of the experiment was 8 weeks. Procedures 1 and 2 included body weight measurement, body composition assessment, blood plasma and urine sampling. EMPA, empagliflozin-treated *db/db* mice; LINA, linagliptin-treated *db/db* mice; EMPA + LINA, empagliflozin–linagliptin-treated *db/db* mice.

The design of the study is presented in Figure 7. After randomization, diabetic *db/db* mice received empagliflozin (Boehringer Inghelheim, Inghelheim, Germany), linagliptin (Boehringer Inghelheim, Inghelheim, Germany), or the combination of both agents, at a dose of 10 mg/kg of body weight diluted in 200 µl of saline. Vehicle-treated mice received only 200 µl. All animals were treated once per day for 56 days from 8 to 16 weeks. Intragastric administration of the studied drugs or vehicle was performed using oral dosing needles 20G x 38 mm curved (pk/3) (VetTech Solutions Ltd, Congleton, UK). The control group included the littermate non-diabetic *db/+* male mice.

Animals were weighed weekly. Body composition was assessed at week 8 and 16 of age by MRI body composition analyzer (Echo Medical Systems, Houston, TX, USA). On the same days, prior to the weighting and body composition study, random blood samples (150–200 μl) were collected from retro-orbital venous sinus using glass Pasteur pipette, previously wetted by heparin 5000 IU/ml. To obtain plasma, blood samples were centrifuged at 3000 g and +4°C for 15 min after collection. Urine samples were collected at week 8 and week 16 of age, prior to the blood sampling. After collection, urine samples were centrifuged at 3000 g and +4°C for 15 min; supernatant was separated and used for the following investigation. The plasma and urine samples were frizzed immediately and stored at –80 °C. At week 16, all mice were sacrificed by decapitation under anesthesia. Kidney samples were obtained for histological assessment, ultrastructural examination, western blot and IHC. To assess renal hypertrophy, kidneys were weighted and adjusted to body weight and lean mass.

### 4.2. Ethical Issues

All experiments were performed in compliance with the protocols and recommendations for the proper use and care of laboratory animals (ECC Directive 86/609/EEC). The protocol was approved by the Ethics Committee of the Institute of Clinical and Experimental Lymphology (Protocol 1/2; 1 April 2014) and by the Inter-Institutional Animal Ethics Committee based on the Institute of Cytology and Genetics SB RAS (Protocol 21; 1 April 2014). All procedures involving animals were reviewed and approved by the Institutional Animal Care and Use Committee of the Center of Genetic Resources of Laboratory Animals based on the SPF Vivarium of Institute of Cytology and Genetics SB RAS (Protocol 246; 8 April 2014).

### 4.3. Laboratory Investigations

The levels of plasma glucose and creatinine were measured on an AU480 Chemical Analyzer (Beckman Coulter, Brea, CA, USA). The levels of fructosamine and glycated albumin in the plasma were determined by ELISA using Mouse Fructosamine (FTA) ELISA kit and Mouse Glycated Albumin (GALB) ELISA kit (MyBioSource, San Diego, CA, USA, catalog number MBS9371486 and MBS733522 respectively). The plasma concentrations of leptin, insulin, ghrelin and PAI-1 were assessed by Multiplex analysis (Bio-Plex Pro™ Mouse Diabetes Multiplex Metabolic Biomarkers Assay, #171F7001M, Bio-Rad Laboratories, Hercules, CA, USA). Urinary albumin and creatinine were measured with a Mouse Albumin ELISA kit and a Mouse Creatinine kit (Crystal Chem, Elk Grove Village, IL, USA, catalog number 80630, 80350); albuminuria was estimated by UACR.

### 4.4. Light Microscopy and IHC

Kidney samples for the light-optical studies and IHC were fixed in 10% formalin (pH = 7.4) for 48 hours, dehydrated in alcohol at increasing concentrations and embedded in Histomix material (BioVitrum, Saint Petersburg, Russia) using a Thermo Scientific™ Citadel 2000 Tissue Processor and Thermo Scientific™ HistoStar™ Embedding Workstation (Fisher Scientific, Pittsburgh, UK). For light-optical studies, sections 5-µm thick were prepared on a microtome LEICA RM2155 (Leica Microsystems GmbH, Wetzlar, Germany) and were stained with Mayer’s hematoxylin and eosin (H&E). Sections were counterstained, dehydrated and mounted.

We assessed glomerular autophagy with staining for beclin-1 and LAMP-1. The formalin-fixed, paraffin-embedded kidney tissue sections on Thermo Scientific™ Polysine Adhesion Slides (Fisher Scientific, Pittsburgh, UK) were dewaxed and rehydrated and submitted to heat-induced epitope retrieval (citrate buffer, pH 6.0). Sections were incubated with Anti-Beclin-1 antibody (1:1000, ab62557, Abcam, Cambridge, UK) or Anti-LAMP1 antibody (1:50, ab25245, Abcam, Cambridge, UK) overnight at +4 °C, washed with PBS three times for 5 min for each wash, incubated with the corresponding secondary antibodies (1/5000; Goat Anti-Rabbit IgG H&L+HRP antibody, ab205718, Abcam, Cambridge, UK and 1/500; Goat Anti-Rat IgG H&L+HRP antibody, ab97057, Abcam, Cambridge, UK) and revealed with DAB substrate kit (ab94665, Abcam, Cambridge, UK). For secondary-antibody-only control, PBST instead of primary antibody was used. Sections were counterstained, dehydrated and mounted.

Light-optical studies were performed with the microscope Axioskop 2 plus (Zeiss, Oberkochen, Germany). The manufacturer’s program equipment was used for acquiring images with size 1300 × 1300 pixels. For acquiring images of IHC, blue filter was additionally used. For analyzing images of H&E stained or IHC slides, 10 glomeruli with maximal section area through the vascular and urinary pole were chosen. Images were morphometried by ImageJ software (Madison, WI, USA). The Vv of mesangium, glomerular beclin-1, and LAMP-1 positive areas were calculated using previously described techniques [66].

### 4.5. TEM

Kidney samples for TEM were fixed in a 4% solution of paraformaldehyde with 0.1 M phosphate buffer (pH = 7.4) followed by 1% OsO_4_. The samples were then dehydrated and embedded in Epon. Kidney sections 70–100 nm thick were contrasted with aqueous uranyl acetate solution and lead citrate and were studied with the JEM-1400 electron microscope (JEOL, Japan).

The width of GBM was measured at 10 points in 10 TEM images of cross-sectioned glomerular barrier on magnification ×100,000 by ImageJ software (Madison, WI, USA). For estimation of mean width and N_A_ of podocyte FPs, 10 images of cross-sectioned glomerular barrier on magnification ×40,000 and ×15,000 respectively were assessed by ImageJ software (Madison, WI, USA).

**Figure 8:**
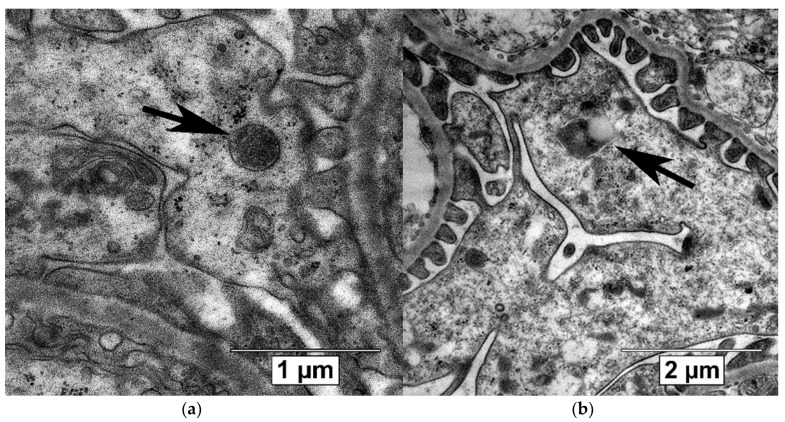
Autophagic compartments in mouse podocytes: lysosomes (black arrow, **a**) and autolysosome (black arrow, **b**). TEM, scale bars: 1 μm (**a**) and 2 µm (**b**).

Autophagic compartments (autophagosomes, autolysosomes and lysosomes) in the podocytes were defined according to the guidelines for the use and interpretation of assay for monitoring autophagy (3^rd^ edition) [27] and linked recommendations [67,68,69]. Autophagosomes were identified as compartments with a double membrane, which is separated by an electron-translucent cleft and encircling cytosol and/or organelles appearing morphologically intact [27,68]. Single-membrane, relatively electron-dense organelles, homogenously filled with very tiny granules, were counted as lysosomes (Figure 8a) [70]. Single-membrane compartments with electron dense cytoplasmic material and/or organelles at various stages of degradation were recognized as autolysosomes (Figure 8b) [27,68]. The Vv of autophagosomes, autolysosomes and lysosomes in the podocyte cytoplasm was assessed on magnification ×40,000 or ×60,000 by previously described methods [66,71] by ImageJ software (Madison, WI, USA).

### 4.6. Westen Blotting

Frozen samples of renal cortex obtained from *db/+* mice and *db/db* mice treated with empagliflozin or empagliflozin–linagliptin were homogenized in protein lysis RIPA buffer (50 mM Tris-HCl pH 8.0, 150 mM NaCl, 1% of Triton X-100, 1% of sodium deoxycholate, 0.1% of SDS, and 1 mM EDTA). The homogenate was centrifuged twice at 14,000× *g* for 30 min at 4 °C to obtain the supernatant. Equal amounts of protein were analyzed by immunoblot assays using anti-LAMP1, anti-Caspase-3, anti-Bcl-2, anti-LC3B antibody (Abcam, Cambridge, UK, ab25245, ab13847, ab692 and ab48394, 1:1000) and β-actin (Abcam, Cambridge, UK, ab1801) as control. Intensities of individual bands were quantified by using ImageJ software (Madison, WI, USA).

### 4.7. Statistical Analysis

Statistical processing was performed using STATISTICA 12 (Dell, Round Rock, TX, USA). Data are presented as medians, minimum and maximum values. Outliers were excluded using Grabbs’ test. The normal distribution was determined by the Kolmogorov–Smirnov test. As most of the variables were not distributed normally, the significance of the differences between independent groups was determined using the non-parametric Mann–Whitney *U*-test. The non-parametrical Wilcoxon test was applied for repeated comparisons. Spearman’s rank correlation analysis was applied to test the association between variables. The differences were considered significant at *p* below 0.05.

## 5. Conclusions

SGLT2 inhibitors and DPP4 inhibitors are relatively new classes of antihyperglycemic agents with distinct renoprotective actions. Identifying the renal effects of these drugs is a challenge to modern medicine. In our study, we demonstrated that both empagliflozin, a SGLT2 inhibitor, and linagliptin, a DPP4 inhibitor, attenuate diabetic kidney disease by promoting glomerular autophagy in *db/db* mice, a model of type 2 diabetes. The acceleration of autophagy flux under empagliflozin and linagliptin treatment is associated with mitigation of glomerular sclerosis, preservation of podocyte morphology, as well as with a slowdown in the growth of albuminuria. Upregulation of beclin-1 and downregulation of caspase-3 could be suggested as possible pathways in autophagy promotion. The data provide a new explanation for the mechanism of the renoprotective action of SGLT2 inhibitors and DPP4 inhibitors in diabetic kidney disease. 

## Figures and Tables

**Table 1:** Body weight and body composition in *db/+* and *db/db* mice.

Parameter	Week of Age	*db/+* Mice (Non-Diabetic Control)	*db/db* Mice			
			Vehicle	EMPA	LINA	EMPA + LINA
*n*		9	10	9	6	6
Body weight, g	8	26.8 (25.0–31.1)	37.1 (32.1–41.6)***	37.4 (28.5–41.5)***	36.5 (29.6–40.3)**	37.3 (35.9–39.5)***
	16	24.8 (21.6–27.7)^+^	37.8 (34.3–40.4)***	52.6 (42.5–62.2)***^##++^	45.3 (39.5–47.5)***^§§+^	50.4 (46.9–52.3)***^#ØØ+^
Fat mass, g	8	3.1 (1.9–4.1)	18.3 (16.2–22.5)***	18.4 (13.5–20.6)***	18.0 (15.1–19.5)***	18.1 (16.7–19.5)***
	16	2.7 (2.1–4.5)	19.6 (17.4–22.7)***	28.0 (22.2–35.3)***^###++^	23.4 (20.3–26.4)***^##+^	26.7 (23.2–28.3)***^###+^
Lean mass, g	8	22.2 (21.0–24.8)	17.1 (16.3–18.7)***	18.2 (13.4–19.8)***	16.0 (13.8–17.2)***	18.5 (15.8–19.1)***
	16	21.2 (19.1–22.5)^+^	16.1 (14.3–22.4)**	21.4 (19.1–24.4)^++^	17.6 (14.9–20.7)**^§+^	20.6 (19.5–21.5)^Ø +^
Total water, g	8	19.5 (18.4–21.5)	14.8 (14.5–16.4)***	15.8 (11.6–17.6)***	12.5 (9.6–14.1)***	16.0 (13.7–16.5)***
	16	18.4 (16.5–19.4)^+^	14.1 (12.3–19.2)*	18.8 (16.7–21.4)^++^	15.5 (13.0–18.1)**^§+^	18.2 (17.3–18.7)^Ø +^

* *p* < 0.05, ** *p* < 0.01, *** *p* < 0.001 vs. non-diabetic control (*db/+*); ^#^ *p* < 0.05, ^##^ *p* < 0.01, ^###^ *p* < 0.001 vs. vehicle-treated *db/db* mice; ^§^ *p* < 0.05, ^§§^ *p* < 0.01 vs. empagliflozin group (Mann–Whitney U-test); ^Ø^ *p* < 0.05, ^ØØ^ *p* < 0.01 vs. linagliptin group (Mann–Whitney U-test); ^+^ *p* < 0.05, ^++^ *p* < 0.01 vs. with week 8 (Wilcoxon test). EMPA, empagliflozin-treated *db/db* mice; LINA, linagliptin-treated *db/db* mice; EMPA+LINA, empagliflozin-linagliptin-treated *db/db* mice. Data are presented as medians (min – max values).

**Table 2:** Plasma biochemical parameters and hormones in *db/db* and *db/+* mice.

Parameter	Week of Age	*db/+* Mice (Non-Diabetic Control)	*db/db* Mice			
			Vehicle	EMPA	LINA	EMPA + LINA
*n*		9	10	9	6	6
Glucose, mmol/L	8	9.5 (5.1–11.4)	28.5 (16.8–40.1)***	23.1 (15.3–35.4)***	27.5 (20.6–48.8)***	25.9 (20.4–33.0)***
	16	9.5 (8.5–12.2)	32.7 (22.5–53.1) ***	16.1 (9.9–23.6)***^###+^	29.9 (25.8–34.6)***^§§^	15.2 (11.7–23.5)***^ØØ##+^
Fructosamine, μmol/L	8	237 (217–249)	456 (424–511)***	480 (425–579)***	436 (383–487)***	430 (406; 475)***
	16	239 (222–296)	622 (524–672)***^++^	468 (341–491)**^+^	650 (591–691)***^§§+^	380 (342–392)*^ØØ+^
Glycated albumin, μmol/L	8	107 (103–127)	227 (206–239)***	235 (217–261)***	209 (156–256)***	217 (203–228)***
	16	117 (109–133)	283 (252–349)***^++^	210 (166–245)**^##+^	315 (225–376)***^§§^	178 (157–182)*^###ØØ+^
Leptin, ng/mL	8	3.30 (1.40–6.54)	97.1 (53.2–114.4)***	93.4 (80.8–133.1)***	92.9 (76.0–118.4)***	89.9 (66.6–119.1)***
	16	3.80 (1.50–6.30)	90.0 (21.2–151.4)***	136.4 (53.0–171.2)***^#+^	96.5 (51.6–172.6)***	139.1 (83.7–200.6)***
Insulin, ng/mL	8	5.59 (3.43–18.0)	25.6 (11.5–45.2)***	21.2 (10.8–40.8)**	22.7 (15.3–54.0)**	22.1 (13.8–76.7)***
	16	10.0 (2.10–22.2)	22.1 (9.8–33.2)**	20.8 (5.95–56.7)*	13.9 (7.2–23.7)	33.3 (13.6–72.9)**^Ø^
Glucagon, ng/mL	8	370 (250–2670)	660 (190–2760)	450 (290–2180)	375 (260–2090)	300 (170–780)
	16	370 (150–2120)	605 (260–2960)	390 (240–860)	325 (190–810)	310 (200–460)^#^
Ghrelin, ng/mL	8	0.99 (0.24–4.27)	0.42 (0.10–1.57)**	0.50 (0.25–1.15)**	0.37 (0.25–0.82)**	0.39 (0.32–1.13)**
	16	1.47 (0.76–6.31)	1.27 (0.57–2.26)*	0.81 (0.28–2.58)	0.45 (0.20–1.14)***^§^	0.77 (0.08–1.47)*
PAI-1, ng/mL	8	1.52 (0.70–3.24)	2.76 (1.41–4.23)*	2.07 (1.00–6.01)	1.84 (0.48–4.20)	2.25 (1.72–3.91)*
	16	3.22 (0.40–4.36)	1.64 (0.09–3.76)	2.22 (0.79–5.12)	1.85 (0.63–4.17)	2.82 (0.98–6.05)

* *p* < 0.05, ** *p* < 0.01, *** *p* < 0.001 vs. non-diabetic control (*db/+*); ^#^ *p* < 0.05, ^##^ *p* < 0.01, ^###^ *p* < 0.001 vs. vehicle-treated *db/db* mice; ^§^ *p* < 0.05, ^§§^ *p* < 0.01 vs. empagliflozin group (Mann–Whitney U-test) ^Ø^ *p* < 0.05, ^ØØ^ *p* < 0.01 vs. linagliptin group (Mann–Whitney U-test); ^+^ *p* < 0.05, ^++^ *p* < 0.01, ^+++^ *p* < 0.001 vs. week 8 (Wilcoxon test). EMPA, empagliflozin-treated *db/db* mice; LINA, linagliptin-treated *db/db* mice; EMPA+LINA, empagliflozin–linagliptin-treated *db/db* mice. PAI-1, plasminogen activator inhibitor-1. Data are presented as medians (min – max values).

**Table 3:** Albuminuria and plasma creatinine in *db/+* and *db/db* mice.

Parameter	Week of Age	*db/+* Mice (Non-Diabetic Control)	*db/db* Mice			
			Vehicle	EMPA	LINA	EMPA + LINA
*n*		9	10	9	6	6
UACR, mg/mmol	8	1.50 (0.60–4.00)	14.8 (10.0–26.9)***	16.3 (12.8–30.0)***	26.8 (19.4–54.7)***	23.5 (14.3–41.4)***
	16	1.80 (0.10–3.10)	21.4 (14.4–29.4)***	11.4 (4.00–22.7)***^###++^	6.9 (4.40–22.4)***^##+^	10.6 (4.50–17.6)***^##+^
Plasma creatinine, μmol/L	8	67.0 (47.0–78.0)	76.4 (70.2–84.2)	70.5 (54.0–82.2)	70.3 (52.8–103.5)	70.4 (66.6– 82.2)
	16	61.8 (54.0–71.7)	75.6 (63.9–89.4)**	81.3 (55.8–91.2)*	71.7 (67.8–99.0)**	80.4 (65.7–91.2)**

* *p* < 0.05, ** *p* < 0.01, *** *p* < 0.001 vs. non-diabetic control (*db/+* mice), Mann–Whitney *U-*test; ^##^ *p* < 0.01, ^###^ *p* < 0.001 vs. vehicle-treated *db/db* mice; ^+^ *p* < 0.05, ^++^ *p* < 0.01 vs. with week 8, Wilcoxon test. EMPA, empagliflozin-treated *db/db* mice; LINA, linagliptin-treated *db/db* mice; EMPA+LINA, empagliflozin–linagliptin-treated *db/db* mice. UACR, urinary albumin-to-creatinine ratio. Data are presented as medians (min - max values).

**Table 4:** Glomerular structural parameters in *db/+* and *db/db* mice.

Parameter	*db/+* Mice (Non-Diabetic Control)	*db/db* Mice			
		Vehicle	EMPA	LINA	EMPA + LINA
*n*	9	10	9	6	6
Mesangial fractional volume, %	14.4 (9.8–18.5)	38.6 (34.5–42.7)***	25.5 (20.4–35.8)**^###^	31.1 (27.3–34.6)**^##^	28.5 (26.1–30.0)**^#^
GBM width, nm	135 (116–157)	163 (135–203)*	139 (116–225)^#^	150 (126–165)^#^	146 (108–152)^#^
Width of podocyte FPs, nm	220 (191–242)	372 (299–426)**	203 (162–317)^##^	189 (172–243)^#^	202 (165–252)^##^
N_A_ of podocyte FPs, nm^–1^	3.39 (3.00–3.79)	2.73 (1.92–3.51)*	3.31 (2.32–4.18)^#^	3.33 (2.85–4.07)^#^	3.25 (2.58–3.56)^#^

* *p* < 0.05, ** *p* < 0.01, *** *p* < 0.001 vs. non-diabetic control (*db/+*); ^#^ *p* < 0.05, ^##^ *p* < 0.01, ^###^ *p* < 0.001 vs. vehicle-treated *db/db* mice; EMPA, empagliflozin-treated *db/db* mice; LINA, linagliptin-treated *db/db* mice; EMPA+LINA, empagliflozin–linagliptin-treated *db/db* mice; GBM, glomerular basement membrane; FPs, foot processes; N_A_, numerical density. Data are presented as medians (min-max values).

**Table 5:** The volume density (Vv) of autophagosomes, autolysosomes and lysosomes in podocytes in *db/+* and *db/db* mice.

Parameter	*db/+* Mice (Non-Diabetic Control)	*db/db* Mice Groups			
		Vehicle	EMPA	LINA	EMPA + LINA
*n*	5	6	5	5	5
Autophagosomes, Vv, %	2.10 (1.80–2.86)	0.80 (0.61–1.68)^***^	2.09 (1.65–2.5)^###^	2.23 (1.73–2.35)^###^	2.14 (1.57–2.31)^###^
Autolysosomes, Vv, %	2.37 (1.85–3.21)	1.09 (0.84–1.69)^***^	2.25 (1.37–3.09)^###^	2.37 (1.58–2.96)^###^	2.22 (1.58–3.04)^###^
Lysosomes, Vv, %	2.94 (2.42–4.00)	2.22 (1.22–2.92)^***^	3.16 (2.06–3.83)^##^	2.64 (2.08–3.52)^#^	2.89 (2.44–3.58)^###^

*** *p* < 0.001 vs. non-diabetic control (*db/+*); ^#^ *p* < 0.05, ^##^ *p* < 0.01, ^###^ *p* < 0.001 vs. vehicle-treated *db/db* mice; EMPA, empagliflozin-treated *db/db* mice; LINA, linagliptin-treated *db/db* mice; EMPA + LINA, empagliflozin–linagliptin-treated *db/db* mice; Vv, volume density. Data are presented as medians (min-max values).

**Table 6:** Relationships between renal autophagy and apoptosis markers and Vv of autophagic compartments in podocytes.

Parameter	Autophagosomes, Vv	Autolysosomes, Vv	Lysosomes, Vv
Glomerular beclin-1 (IHC)	*r* = 0.58 *p* = 0.0001, *n* = 26	*r* = 0.48 *p* = 0.002, *n* = 26	*r* = 0.25 *p* > 0.05, *n* = 26
Glomerular LAMP-1 (IHC)	*r* = 0.46 *p* = 0.003, *n* = 26	*r* = 0.50 *p* = 0.001, *n* = 26	*r* = 0.42 *p* = 0.008, *n* = 26
Renal LC3B (WB)	*r* = −0.57 *p* = 0.009, *n* = 20	*r* = −0.69 *p* = 0.0008, *n* = 20	*r* = −0.55 *p* = 0.01, *n* = 20
Renal LAMP-1 (WB)	*r* = 0.50 *p* = 0.02, *n* = 20	*r* = 0.55 *p* = 0.01, *n* = 20	*r* = 0.63 *p* = 0.003, *n* = 20
Renal caspase-3 (WB)	*r* = −0.35 *p* > 0.05, *n* = 20	*r* = −0.56 *p* = 0.01, *n* = 20	*r* = −0.44 *p* > 0.05, *n* = 20
Renal Bcl-2 (WB)	*r* = 0.54 *p* = 0.01, *n* = 20	*r* = 0.57 *p* = 0.009, *n* = 20	*r* = 0.63 *p* = 0.003, *n* = 20

Data are presented as Spearman’s rank correlation coefficients, *p*-value and number of observations; IHC, immunohistochemistry; Vv, volume density; WB, western blot.

**Table 7:** Relationships between glomerular and podocyte autophagy characteristics and other parameters of renal morphology.

Parameter	Glomerular beclin-1, %	Glomerular LAMP-1, %	Autophagosomes in Podocytes, Vv	Autolysosomes in Podocytes, Vv	Lysosomes in Podocytes, Vv
Kidneys weight/lean mass ratio	*r* = −0.13 *p* > 0.05, *n* = 33	*r* = −0.19 *p* > 0.05, *n* = 33	*r* = −0.31 *p* > 0.05, *n* = 26	*r* = −0.43 *p* = 0.007, *n* = 26	*r* = −0.45 *p* = 0.007, *n* = 26
Mesangial fractional volume	*r* = −0.39 *p* = 0.02, *n* = 40	*r* = −0.37 *p* = 0.03, *n* = 40	*r* = −0.36 *p* = 0.03, *n* = 40	*r* = −0.50 *p* = 0.002, *n* = 40	*r* = −0.32 *p* = 0.009, *n* = 40
Mean width of GBM	*r* = −0.41 *p* = 0.03, *n* = 26	*r* = −0.40 *p* = 0.03, *n* = 26	*r* = −0.54 *p* = 0.003, *n* = 26	*r* = −0.45 *p* = 0.01, *n* = 26	*r* = −0.26 *p* > 0.05, *n* = 26
Mean width of podocyte FPs	*r* = −0.62 *p* = 0.0004, *n* = 26	*r* = −0.29 *p* > 0.05, *n* = 26	*r* = −0.58 *p* = 0.0009, *n* = 26	*r* = −0.49 *p* = 0.007, *n* = 26	*r* = −0.41 *p* = 0.03, *n* = 26
N_A_ of podocyte FPs	*r* = 0.27 *p* > 0.05, *n* = 26	*r* = 0.32 *p* > 0.05, *n* = 26	*r* = 0.54 *p* = 0.003, *n* = 26	*r* = 0.47 *p* = 0.01, *n* = 26	*r* = 0.35 *p* > 0.05, *n* = 26

Data are presented as Spearman’s rank correlation coefficients; GBM, glomerular basement membrane; FPs, foot processes; N_A_, numerical density; Vv, volume density

**Table 8:** Relationships between expression of autophagy and apoptosis regulators in the renal cortex and parameters of renal and glomerular morphology.

Parameter	Renal LC3B	Renal LAMP-1	Renal Caspase-3	Renal Bcl-2
Kidney weight/lean mass ratio	*r* = 0.40 *p* > 0.05	*r* = −0.60 *p* = 0.005	*r* = 0.64 *p* = 0.002	*r* = −0.64 *p* = 0.002
Mesangial fractional volume	*r* = 0.45 *p* = 0.045	*r* = −0.71 *p* = 0.0004	*r* = 0.67 *p* = 0.001	*r* = −0.71 *p* = 0.0004
Mean width of GBM	*r* = 0.67 *p* = 0.002	*r* = −0.45 *p* = 0.045	*r* = 0.69 *p* = 0.002	*r* = −0.53 *p* = 0.02
Mean width of podocyte FPs	*r* = 0.56 *p* = 0.02	*r* = −0.38 *p* > 0.05	*r* = 0.52 *p* = 0.02	*r* = −0.44 *p* = 0.048
N_A_ of podocyte FPs	*r* = −0.46 *p* = 0.04	*r* = 0.38 *p* > 0.05	*r* = −0.62 *p* = 0.004	*r* = 0.46 *p* = 0.04

Data are presented as Spearman’s rank correlation coefficients. *n* = 20. GBM, glomerular basement membrane; FPs, foot processes; N_A_, numerical density; Vv, volume density

## Abbreviations

AMPK	AMP-activated protein kinase
DPP4	Dipeptidylpeptidase-4
GLUT4	Gucose transporter type 4
IHC	Immunohistochemistry
mTOR	mammalian Target of rapamycin
N_A_	Numerical density
LAMP-1	Lysosomal-associated membrane protein-1
PAI-1	Plasminogen activator inhibitor-1
PFs	Foot processes
SGLT2	Sodium-glucose cotransporter-2
TEM	Transmission electron microscopy
UACR	Urinary albumin-to-creatinine ratio
Vv	Volume density

## References

[1] Webster A.C., Nagler E.V., Morton R.L., Masson P. (2017). Chronic kidney disease. Lancet.

[2] Lin J.S., Susztak K. (2016). Podocytes: The weakest link in diabetic kidney disease?. Curr. Diab. Rep..

[3] Maestroni S., Zerbini G. (2018). Glomerular endothelial cells versus podocytes as the cellular target in diabetic nephropathy. Acta Diabetol..

[4] Yasuda-Yamahara M., Kume S., Tagawa A., Maegawa H., Uzu T. (2015). Emerging role of podocyte autophagy in the progression of diabetic nephropathy. Autophagy.

[5] Liu N., Xu L., Shi Y., Zhuang S. (2017). Podocyte autophagy: A potential therapeutic target to prevent the progression of diabetic nephropathy. J. Diabetes Res..

[6] Lin T.A., Wu V.C., Wang C.Y. (2019). Autophagy in chronic kidney diseases. Cells.

[7] Nagata M. (2016). Podocyte injury and its consequences. Kidney Int..

[8] Yang D., Livingston M.J., Liu Z., Dong G., Zhang M., Chen J.K., Dong Z. (2018). Autophagy in diabetic kidney disease: Regulation, pathological role and therapeutic potential. Cell Mol. Life Sci..

[9] Zhang Y., Whaley-Connell A.T., Sowers J.R., Ren J. (2018). Autophagy as an emerging target in cardiorenal metabolic disease: From pathophysiology to management. Pharmacol. Ther..

[10] Wanner C., Inzucchi S.E., Lachin J.M., Fitchett D., von Eynatten M., Mattheus M., Johansen O.E., Woerle H.J., Broedl U.C., Zinman B. (2016). Empagliflozin and progression of kidney disease in type 2 diabetes. N. Engl. J. Med..

[11] Rosenstock J., Perkovic V., Johansen O.E., Cooper M.E., Kahn S.E., Marx N., Alexander J.H., Pencina M., Toto R.D., Wanner C. (2019). Effect of linagliptin vs placebo on major cardiovascular events in adults with type 2 diabetes and high cardiovascular and renal risk: The CARMELINA randomized clinical trial. JAMA.

[12] Birnbaum Y., Bajaj M., Yang H.C., Ye Y. (2018). Combined SGLT2 and DPP4 inhibition reduces the activation of the Nlrp3/ASC inflammasome and attenuates the development of diabetic nephropathy in mice with type 2 diabetes. Cardiovasc. Drugs Ther..

[13] Zou H., Zhou B., Xu G. (2017). SGLT2 inhibitors: A novel choice for the combination therapy in diabetic kidney disease. Cardiovasc. Diabetol..

[14] Fioretto P., Zambon A., Rossato M., Busetto L., Vettor R. (2016). SGLT2 inhibitors and the diabetic kidney. Diabetes Care.

[15] Korbut A.I., Klimontov V.V. (2017). Empagliflozin: A new strategy for nephroprotection in diabetes. Diabetes Mellit..

[16] Nespoux J., Vallon V. (2018). SGLT2 inhibition and kidney protection. Clin. Sci. (Lond.).

[17] Korbut A.I., Klimontov V.V. (2016). Incretin-based therapy: Renal effects. Diabetes Mellit..

[18] Scheen A.J., Delanaye P. (2018). Renal outcomes with dipeptidyl peptidase-4 inhibitors. Diabetes Metab..

[19] Gupta S., Sen U. (2019). More than just an enzyme: Dipeptidyl peptidase-4 (DPP-4) and its association with diabetic kidney remodelling. Pharmacol. Res..

[20] Lee Y.H., Kim S.H., Kang J.M., Heo J.H., Dong-Jin K., Seon H.P., Min J.S., Jaehee K., Jisu O., Dong H.Y. (2019). Empagliflozin attenuates diabetic tubulopathy by improving mitochondrial fragmentation and autophagy. Am. J. Physiol. Renal Physiol..

[21] Yang C.C., Chen Y.T., Wallace C.G., Chen K.H., Cheng B.C., Sung P.H., Li Y.C., Ko S.F., Chang H.W., Yip H.K. (2019). Early administration of empagliflozin preserved heart function in cardiorenal syndrome in rat. Biomed. Pharmacother..

[22] Ashrafizadeh M., Yaribeygi H., Atkin S.L., Sahebkar A. (2019). Effects of newly introduced antidiabetic drugs on autophagy. Diabetes Metab. Syndr..

[23] Li X.Y., Wang S.S., Han Z., Han F., Chang Y.P., Yang Y., Xue M., Sun B., Chen L.M. (2017). Triptolide restores autophagy to alleviate diabetic renal fibrosis through the miR-141-3p/PTEN/Akt/mTOR Pathway. Mol. Ther. Nucleic Acids..

[24] Xin W., Li Z., Xu Y., Yu Y., Zhou Q., Chen L., Wan Q. (2016). Autophagy protects human podocytes from high glucose-induced injury by preventing insulin resistance. Metabolism.

[25] Tagawa A., Yasuda M., Kume S., Yamahara K., Nakazawa J., Chin-Kanasaki M., Araki H., Araki S., Koya D., Asanuma K. (2016). Impaired podocyte autophagy exacerbates proteinuria in diabetic nephropathy. Diabetes.

[26] Liu Y., Zhang J., Wang Y., Zeng X. (2017). Apelin involved in progression of diabetic nephropathy by inhibiting autophagy in podocytes. Cell Death Dis..

[27] Klionsky D.J., Abdelmohsen K., Abe A., Abedin M.J., Abeliovich H., Acevedo Arozena A., Adachi H., Adams C.M., Adams P.D., Adeli K. (2016). Guidelines for the use and interpretation of assays for monitoring autophagy (3rd edition). Autophagy.

[28] Runwal G., Stamatakou E., Siddiqi F.H., Puri C., Zhu Y., Rubinsztein D.C. (2019). LC3-positive structures are prominent in autophagy-deficient cells. Sci. Rep..

[29] Xu C., Wang W., Zhong J., Lei F., Xu N., Zhang Y., Xie W. (2018). Canagliflozin exerts anti-inflammatory effects by inhibiting intracellular glucose metabolism and promoting autophagy in immune cells. Biochem. Pharmacol..

[30] Gong J., Zhan H., Li Y., Zhang W., Jin J., He Q. (2019). Krüppel-like factor 4 ameliorates diabetic kidney disease by activating autophagy via the mTOR pathway. Mol. Med. Rep..

[31] Lee E.J., Kang M.K., Kim Y.H., Kim D.Y., Oh H., Kim S.I., Oh S.Y., Kang Y.H. (2019). Dietary chrysin suppresses formation of actin cytoskeleton and focal adhesion in AGE-exposed mesangial cells and diabetic kidney: Role of autophagy. Nutrients.

[32] Dong C., Zheng H., Huang S., You N., Xu J., Ye X., Zhu Q., Feng Y., You Q., Miao H. (2015). Heme oxygenase-1 enhances autophagy in podocytes as a protective mechanism against high glucose-induced apoptosis. Exp. Cell Res..

[33] Guo H., Wang B., Li H., Ling L., Niu J., Gu Y. (2018). Glucagon-like peptide-1 analog prevents obesity-related glomerulopathy by inhibiting excessive autophagy in podocytes. Am. J. Physiol. Renal Physiol..

[34] Zhao X., Liu G., Shen H., Gao B., Li X., Fu J., Zhou J., Ji Q. (2015). Liraglutide inhibits autophagy and apoptosis induced by high glucose through GLP-1R in renal tubular epithelial cells. Int. J. Mol. Med..

[35] Feng Y., Chen S., Xu J., Zhu Q., Ye X., Ding D., Yao W., Lu Y. (2018). Dysregulation of lncRNAs GM5524 and GM15645 involved in high-glucose-induced podocyte apoptosis and autophagy in diabetic nephropathy. Mol. Med. Rep..

[36] Lenoir O., Jasiek M., Hénique C., Guyonnet L., Hartleben B., Bork T., Chipont A., Flosseau K., Bensaada I., Schmitt A. (2015). Endothelial cell and podocyte autophagy synergistically protect from diabetes-induced glomerulosclerosis. Autophagy.

[37] Lim J.H., Kim H.W., Kim M.Y., Kim T.W., Kim E.N., Kim Y., Chung S., Kim Y.S., Choi B.S., Kim Y.S. (2018). Cinacalcet-mediated activation of the CaMKKβ-LKB1-AMPK pathway attenuates diabetic nephropathy in *db/db* mice by modulation of apoptosis and autophagy. Cell Death Dis..

[38] Inoki K. (2014). mTOR signaling in autophagy regulation in the kidney. Semin. Nephrol..

[39] De Rechter S., Decuypere J.P., Ivanova E., van den Heuvel L.P., De Smedt H., Levtchenko E., Mekahli D. (2016). Autophagy in renal diseases. Pediatr. Nephrol..

[40] Zhou L., Liu Y. (2015). Wnt/β-catenin signalling and podocyte dysfunction in proteinuric kidney disease. Nat. Rev. Nephrol..

[41] Ding Y., Choi M.E. (2014). Regulation of autophagy by TGF-β: Emerging role in kidney fibrosis. Semin. Nephrol..

[42] Dai H., Liu Q., Liu B. (2017). Research progress on mechanism of podocyte depletion in diabetic nephropathy. J. Diabetes Res..

[43] Kitada M., Ogura Y., Monno I., Koya D. (2017). Regulating autophagy as a therapeutic target for diabetic nephropathy. Curr. Diab. Rep..

[44] Packer M. (2020). Interplay of adenosine monophosphate-activated protein kinase/sirtuin-1 activation and sodium influx inhibition mediates the renal benefits of sodium-glucose co-transporter-2 inhibitors in type 2 diabetes: A novel conceptual framework. Diabetes Obes. Metab..

[45] Zhao X., Chen Y., Tan X., Zhang L., Zhang H., Li Z., Liu S., Li R., Lin T., Liao R. (2018). Advanced glycation end-products suppress autophagic flux in podocytes by activating mammalian target of rapamycin and inhibiting nuclear translocation of transcription factor EB. J. Pathol..

[46] Shi M., Yang S., Zhu X., Sun D., Sun D., Jiang X., Zhang C., Wang L. (2019). The RAGE/STAT5/autophagy axis regulates senescence in mesangial cells. Cell Signal..

[47] Malik S.A., Mariño G., BenYounès A., Shen S., Harper F., Maiuri M.C., Kroemer G. (2011). Neuroendocrine regulation of autophagy by leptin. Cell Cycle.

[48] Wang B., Chandrasekera P.C., Pippin J.J. (2014). Leptin- and leptin receptor-deficient rodent models: Relevance for human type 2 diabetes. Curr. Diabetes Rev..

[49] Audzeyenka I., Rogacka D., Piwkowska A., Angielski S., Jankowski M. (2017). Viability of primary cultured podocytes is associated with extracellular high glucose-dependent autophagy downregulation. Mol. Cell Biochem..

[50] Coward R., Fornoni A. (2015). Insulin signaling: Implications for podocyte biology in diabetic kidney disease. Curr. Opin. Nephrol. Hypertens..

[51] Guzman J., Jauregui A.N., Merscher-Gomez S., Maiguel D., Muresan C., Mitrofanova A., Diez-Sampedro A., Szust J., Yoo T.H., Villarreal R. (2014). Podocyte-specific GLUT4-deficient mice have fewer and larger podocytes and are protected from diabetic nephropathy. Diabetes.

[52] Ezquerro S., Frühbeck G., Rodríguez A. (2017). Ghrelin and autophagy. Curr. Opin. Clin. Nutr. Metab. Care.

[53] Wang Z.H., Ren W.Y., Zhu L., Hu L.J. (2014). Plasminogen activator inhibitor-1 regulates LPS induced inflammation in rat macrophages through autophagy activation. Sci. World J..

[54] Hsu Y.H., Chuang H.C., Lee Y.H., Lin Y.F., Chiu Y.J., Wang Y.L., Wu M.S., Chiu H.W. (2019). Induction of fibrosis and autophagy in kidney cells by vinyl chloride. Cells.

[55] Kanasaki K., Kawakita E., Koya D. (2019). Relevance of Autophagy Induction by Gastrointestinal Hormones: Focus on the Incretin-Based Drug Target and Glucagon. Front. Pharmacol..

[56] Kaushal G.P., Chandrashekar K., Juncos L.A., Shah S.V. (2020). Autophagy function and regulation in kidney disease. Biomolecules.

[57] Zhu Y., Zhao L., Liu L., Gao P., Tian W., Wang X., Jin H., Xu H., Chen Q. (2010). Beclin 1 cleavage by caspase-3 inactivates autophagy and promotes apoptosis. Protein Cell.

[58] Mizuno M., Kuno A., Yano T., Miki T., Oshima H., Sato T., Nakata K., Kimura Y., Tanno M., Miura T. (2018). Empagliflozin normalizes the size and number of mitochondria and prevents reduction in mitochondrial size after myocardial infarction in diabetic hearts. Physiol. Rep..

[59] Zhou Y., Wang H., Man F., Guo Z., Xu J., Yan W., Li J., Pan Q., Wang W. (2018). Sitagliptin protects cardiac function by reducing nitroxidative stress and promoting autophagy in Zucker diabetic fatty (ZDF) rats. Cardiovasc. Drugs Ther..

[60] Murase H., Kuno A., Miki T., Tanno M., Yano T., Kouzu H., Ishikawa S., Tobisawa T., Ogasawara M., Nishizawa K. (2015). Inhibition of DPP-4 reduces acute mortality after myocardial infarction with restoration of autophagic response in type 2 diabetic rats. Cardiovasc. Diabetol..

[61] Gu Y., Ma C.T., Gu H.L., Shi L., Tian X.T., Xu W.Q. (2018). Sitagliptin improves cardiac function after myocardial infarction through activation of autophagy in streptozotocin-induced diabetic mice. Eur. Rev. Med. Pharmacol. Sci..

[62] Zheng W., Zhou J., Song S., Kong W., Xia W., Chen L., Zeng T. (2018). Dipeptidyl-peptidase 4 inhibitor sitagliptin ameliorates hepatic insulin resistance by modulating inflammation and autophagy in *ob/ob* mice. Int. J. Endocrinol..

[63] Ashrafi Jigheh Z., Ghorbani Haghjo A., Argani H., Roshangar L., Rashtchizadeh N., Sanajou D., Nazari Soltan Ahmad S., Rashedi J., Dastmalchi S., Mesgari Abbasi M. (2019). Empagliflozin alleviates renal inflammation and oxidative stress in streptozotocin-induced diabetic rats partly by repressing HMGB1-TLR4 receptor axis. Iran. J. Basic Med. Sci..

[64] Sharkovska Y., Reichetzeder C., Alter M., Tsuprykov O., Bachmann S., Secher T., Klein T., Hocher B. (2014). Blood pressure and glucose independent renoprotective effects of dipeptidyl peptidase-4 inhibition in a mouse model of type-2 diabetic nephropathy. J. Hypertens..

[65] Gavrilova Y.S., Bgatova N.P., Klimontov V.V., Ischenko I.Y., Michurina S.V., Myakina N.E., Zavyalov E.L. (2016). Effect of linagliptin on structural changes in the kidney in experimental type 2 diabetes mellitus. Bull. Exp. Biol. Med..

[66] Mandarim-de-Lacerda C.A., Del Sol M. (2017). Tips for studies with quantitative morphology (morphometry and stereology). Int. J. Morphol..

[67] Eskelinen E.L. (2008). To be or not to be? Examples of incorrect identification of autophagic compartments in conventional transmission electron microscopy of mammalian cells. Autophagy.

[68] Yoshii S.R., Mizushima N. (2017). Monitoring and measuring autophagy. Int. J. Mol. Sci..

[69] Higgins G.C., Nguyen T.V., Ramm G., Coughlan M.T. (2018). Methods in renal research: Measurement of autophagic flux in the renal cortex ex vivo. Nephrology (Carlton).

[70] Lodish H., Berk A., Zipursky S.L., Matsudaira P., Baltimore D., Darnell J., Freeman W.H. (2000). Molecular Cell Biology.

[71] Cheville N.F., Stasko J. (2014). Techniques in electron microscopy of animal tissue. Vet. Pathol..

